# Factors Associated with Omission of Axillary Lymph Node Dissection for Patients with Clinical Node-Positive Breast Cancer Who have Residual Nodal Disease on Pathology After Neoadjuvant Chemotherapy

**DOI:** 10.1245/s10434-026-19968-5

**Published:** 2026-06-26

**Authors:** Nicole Rademacher, Lily A. Gutnik, Kristy K. Broman

**Affiliations:** 1 Department of Surgery, University of Alabama at Birmingham, Birmingham, AL USA; 2 Institute for Cancer Outcomes and Survivorship, University of Alabama at Birmingham, Birmingham, AL USA; 3 The University of Texas MD Anderson Cancer Center, Houston, TX USA

**Keywords:** Breast cancer, Axillary surgery, Node-positive breast cancer, Residual axillary disease, De-escalation

## Abstract

**Background:**

For breast cancer patients with clinically node-positive (cN+) and residual disease after neoadjuvant chemotherapy (NAC), axillary lymph node dissection (ALND) remains the standard of care, though alternative strategies may be selected by multidisciplinary teams in certain cases. This study evaluated factors associated with omission of ALND for cN+ patients who were pathologically node-positive after NAC (ypN+).

**Methods:**

In this retrospective observational study, the National Cancer Database was queried for patients with clinical T1–T4, N1–N3 breast cancer from 2016 to 2021 who received NAC and were subsequently ypN+. Axillary procedures were categorized as sentinel lymph node biopsy (SLNB) alone, SLNB followed by ALND (SLNB + ALND), or ALND alone. Logistic regression identified factors associated with omission of ALND after SLNB, adjusting for sociodemographic, geographic, and clinical variables.

**Results:**

Among 38,559 patients, axillary interventions were SLNB alone (*n* = 7,131, 18.49%), SLNB+ALND (*n* = 14,247, 36.95%), and ALND alone (*n* = 17,179, 44.55%). Over the study period, SLNB alone and SLNB + ALND increased while ALND alone decreased. Compared with academic programs, patients treated at comprehensive community, integrated network, and community programs had greater odds of SLNB alone compared with SLNB+ALND (odds ratio [OR] 1.32, 95% confidence interval [CI] 1.22–1.43; OR 1.17, 95% CI 1.07–1.27; OR 1.54, 95% CI 1.33–1.78, respectively).

**Conclusions:**

There has been de-escalation of ALND after initial SLNB for cN+ breast cancer patients found to be ypN+. This suggests that providers may be extrapolating evidence supporting SLNB alone to populations in which it has not yet been thoroughly studied or are omitting ALND in anticipation of ongoing clinical trial results.

**Supplementary Information:**

The online version contains supplementary material available at https://doi.org/10.1245/s10434-026-19968-5.

Over the past two decades, surgical management of breast cancer, and management of the axilla in particular, has evolved substantially, driven in part by growing evidence supporting de-escalation of axillary surgery.^1^ Several studies, including ACOSOG Z0011, AMAROS, and SENOMAC, in patients who did not receive neoadjuvant chemotherapy (NAC), as well as ACOSOG Z1071, SENTINA, GANEA2, and SN FNAC in patients with clinically node-positive disease who received NAC, have supported tailored approaches to axillary management in which axillary lymph node dissection (ALND) may be safely de-implemented in select patients.^2–9^

For clinically node positive (cN+) patients who receive NAC, historical surgical standard of care has been ALND. However, recent studies have shown that in select patients with cN+ breast cancer who convert to clinically node negative (cN0) after NAC, sentinel lymph node biopsy (SLNB) or SLNB with removal of the marked/clipped node (targeted axillary dissection [TAD]) are appropriate and can be used for staging and definitive axillary surgery for those achieving ypN0.^10–14^ Currently, for patients with cN+ disease who have residual axillary disease after NAC, ALND remains the standard of care.^15,16^

When a high degree of de-implementation occurs, as was evidenced by the change in ALND rates after the dissemination of the Z0011 trial results, practices, such as ALND, may be omitted in populations for which it has not yet been studied or remains an active area of ongoing research.^17^ Prior work demonstrated variable de-implementation of cancer care evidence, even among centers accredited by the Commission on Cancer (CoC), with differences observed by facility type (e.g., Comprehensive Community Program versus Community Program).^18,19^ Understanding the unintended consequences of de-implementation, in particular the extrapolation of evidence to populations for which the practice has not been thoroughly studied, and which practice locations are at risk, is critical to helping assure evidence-based care is appropriately applied.^20^ In this study, we sought to evaluate factors associated with omission of ALND after SLNB among patients with residual nodal disease after neoadjuvant chemotherapy.

## Methods

### Study Design, Setting, and Participants

**Fig. 1:**
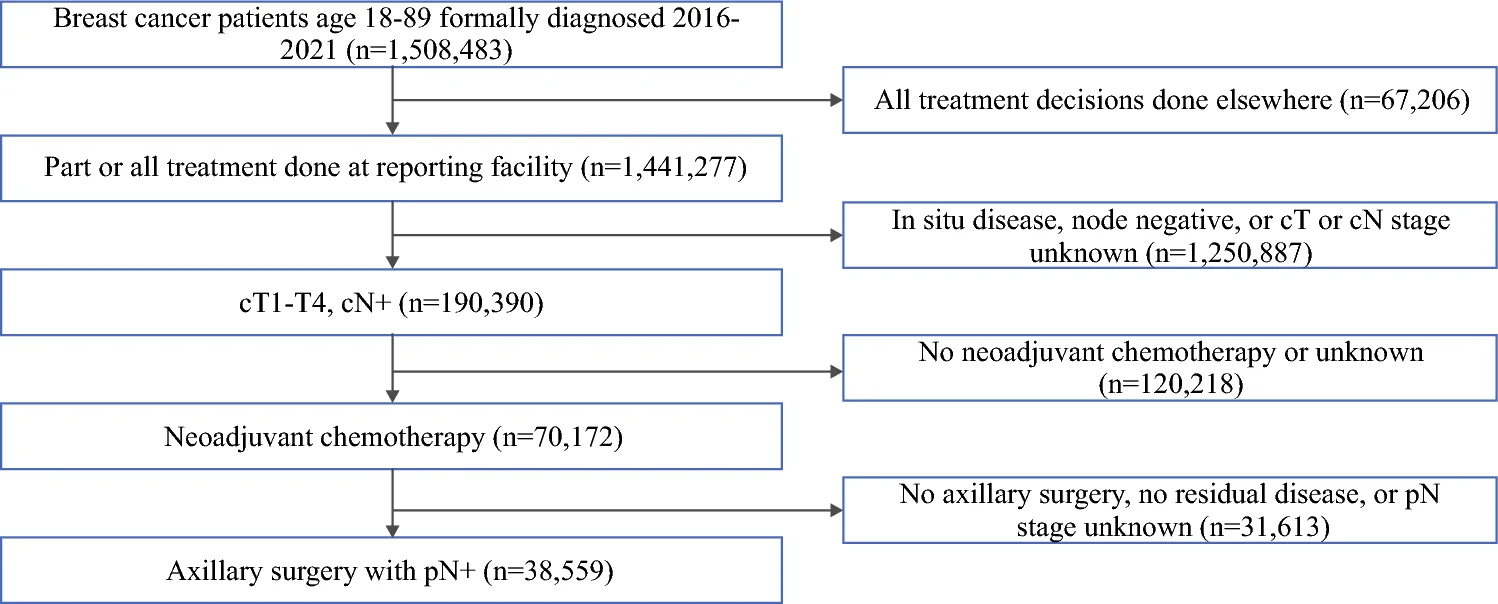
Cohort creation

This was a retrospective observational study using 2016-2021 National Cancer Database (NCDB) data to evaluate factors associated with omission of ALND after initial SLNB for cN+ patients found to be ypN+. The study was exempt from Institutional Review Board review given use of a de-identified dataset. The NCDB Participant User File for breast cancer was queried for patients formally diagnosed between 2016 and 2021 who had part or all of their treatment done at the reporting facility. This period was chosen because it follows publication of SENTINA, ACOSOG Z1071, and SN FNAC, all of which may have contributed to de-escalation of ALND after NAC, and because it was the most recent data available at the time of analysis. The cohort was restricted to patients < 90 years with clinically staged T1–T4 (cT1–cT4), N1–N3 (cN1–cN3) breast cancer who received NAC and had residual nodal disease (pathologic node positive) on axillary surgery (ypN+) (Fig. 1). Patients with isolated tumor cells (itc+) or were molecularly positive (mol+) were classified pathologically node negative (pN0) and thus excluded. Clinical nodal status between NAC and surgery was unknown due to limitations in available data. The manuscript is reported according to STROBE guidelines for observational cohort studies (supplemental materials).

### Primary Outcome and Exposure

The primary outcome was type of axillary surgery. This was categorized into three groups: SLNB alone, SLNB followed by ALND (SLNB+ALND), and ALND alone. The primary exposure of interest was CoC facility type (Academic/Research Program, Comprehensive Community Cancer Program, Community Cancer Program, Integrated Network Cancer Program).

### Secondary Exposures

Patient sociodemographic characteristics included age, sex, race and ethnicity, Charlson–Deyo comorbidity score, and insurance status. Area level, zip-code based variables included education, defined as percentage without a high school degree (< 5.0%, 5–9%, 9.1–5.2%, ≥ 15.3%+), and median household income (< $46,277, $46,277–$57,856, $57,857–$74,062, and ≥ $74,063), which were categorized into quartiles within the NCDB. Geographic characteristics included distance traveled and rurality. Distance traveled was defined as crowfly distance and was categorized into three groups: < 10 miles, 10–30 miles, and > 30 miles.^21^ Rurality was defined using the Rural-Urban Continuum Code (RUCC) with 1–3 classified as metropolitan and 4–9 classified as rural. Breast cancer disease and treatment characteristics included clinical T and clinical N stage, pathologic N stage, hormone receptor (HR) status, human epidermal growth factor-2 (HER2) receptor status, type of breast surgery, breast radiation, and nodal radiation. Breast surgery was categorized as mastectomy or partial mastectomy. Year of diagnosis was treated as a continuous variable in regression models (2016–2021).

### Statistical Analysis

Descriptive statistics were calculated. Categorical variables are described as frequencies with percentages. Bivariate analyses were performed to evaluate cohort characteristics by axillary surgery type using a chi-square test or Kruskal–Wallis test as appropriate. A multivariable logistic regression model was created to evaluate the odds of omission of ALND after receiving SLNB alone and was adjusted for sociodemographic, geographic, and clinical characteristics. Additional subgroup analyses were performed by pN stage (pN1, pN2, and pN3) to examine whether time and facility type effects were consistent across degree of pathologic nodal involvement and to identify influential factors within pathologic nodal stage groups. Observations with missing or unknown values were retained as a separate category and included in models as such. Results are reported as odds ratios (OR) with 95% confidence intervals (95% CI). Statistical analyses were conducted in Stata Version 18.0 (StataCorp, College Station, TX).

## Results

### Cohort Descriptives

A total of 1,508,483 cases of breast cancer diagnosed between 2016 and 2021 among patients aged 18–89 were identified (Fig. 1). Patients with all treatment decisions made elsewhere (*n* = 67,206) were excluded. An additional 1,250,887 patients were excluded for in-situ disease, node negative disease, or unknown cT or cN stage. Another 120,218 patients were excluded for no or unknown receipt of NAC. Lastly, patients who did not undergo axillary surgery or did not have a reported pN stage were excluded. There were 38,559 remaining patients with clinical T1–T4 node positive breast cancer who underwent NAC followed by axillary surgery (SLNB alone: n = 7,131, 18.49%; SLNB + ALND: n = 14,247, 36.95%; ALND alone: *n* = 17,179, 44.55%) and were found to have residual disease on pathology (Table 1). The most common facility types for treatment were comprehensive community programs (*n* = 12,455, 32.3%) and academic/research programs (*n* = 11,691, 30.32%), followed by integrated network programs (*n* = 7,396, 19.18%) and community programs (*n* = 2,050, 5.32%) with the remainder being unknown (*n* = 4,967, 12.88%). The overall cohort was predominantly female (*n* = 38,217, 99.11%) and non-Hispanic White (*n* = 24,719, 64.11%) with a median age of 55 years interquartile range (IQR) 46, 64]. Most patients were otherwise healthy (Charlson–Deyo Comorbidity Index of 0: *n* = 32,329, 83.84%). In regard to socioeconomic characteristics, the majority of patients were privately insured (*n* = 22,293, 57.82%) and lived in areas where the median household income is > $74,062 and where most people had at least a high school degree (*n* = 25,625, 66.46%). Patients tended to reside in metropolitan areas (*n* = 33,007, 85.60%) and travel less than 10 miles to care (*n* = 15,793, 40.95%).

**Table 1:** Overall cohort descriptives

Characteristic	Cohort (*N* = 38,559) *n* (%)
CoC cancer program facility type^a^	
Academic	11,691 (30.32)
Comprehensive community	12,455 (32.30)
Integrated network	7396 (19.18)
Community	2050 (5.32)
Unknown	4967 (12.88)
Year of diagnosis	
2016	6138 (15.92)
2017	6018 (15.61)
2018	6345 (16.46)
2019	6618 (17.16)
2020	6512 (16.89)
2021	6928 (17.97)
Age (years)	
Median, IQR	55 (46, 64)
Sex	
Female	38,217 (99.11)
Male	342 (0.89)
Race	
Non-Hispanic White	24,719 (64.11)
Non-Hispanic Black	7086 (18.38)
Hispanic	3924 (10.18)
Non-Hispanic Asian	1779 (4.61)
Non-Hispanic American Indian or Alaskan Native	177 (0.46)
Non-Hispanic Native Hawaiian or Pacific Islander	145 (0.38)
Other or unknown	729 (1.89)
Charlson–Deyo Comorbidity Index	
0	32,329 (83.84)
1	4523 (11.73)
2	1048 (2.72)
3+	659 (1.71)
Insurance	
Private	22,293 (57.82)
Medicare	9250 (23.99)
Medicaid	4882 (12.66)
Uninsured	1134 (2.94)
Other government	621 (1.61)
Unknown	379 (0.98)
Median income	
< $46,227	5545 (14.38)
$46,227–$57,856	6780 (17.58)
$57,857–$74,062	7631 (19.79)
≥ $74,063	12,676 (32.87)
Unknown	5927 (15.37)
Education	
< 5% with no HSD	7070 (18.34)
5–9% with no HSD	9336 (24.21)
9.1–15.2% with no HSD	9012 (23.37)
≥ 15.3% with no HSD	7277 (18.87)
Unknown	5864 (15.21)
Rurality^b^	
Metropolitan	33,007 (85.60)
Rural	4594 (11.91)
Unknown	958 (2.48)
Distance traveled (miles)	
< 10	15,793 (40.96)
10–30	11,211 (29.10)
> 30	5951 (15.43)
Unknown	5594 (14.51)
Clinical T stage	
cT1	6215 (16.12)
cT2	17,784 (46.12)
cT3	8777 (22.76)
cT4	5783 (15.00)
Clinical N stage	
cN1	30,789 (79.85)
cN2	4222 (10.95)
cN3	3548 (9.20)
Pathologic N stage	
pN1	23,108 (59.93)
pN2	10,648 (27.61)
pN3	4803 (12.46)
Hormone receptor status	
HR+	27,543 (71.43)
HR–	10,890 (28.24)
Unknown	126 (0.33)
HER2 status	
HER2+	3638 (9.43)
HER2−	34,226 (88.76)
Unknown	695 (1.80)
Axillary surgery	
SLNB	7137 (18.51)
SLNB + ALND	14,249 (36.95)
ALND	17,173 (44.54)
Breast surgery	
Mastectomy	26,897 (69.76)
Partial mastectomy	11,311 (29.33)
Unknown	351 (0.91)
Breast radiation	
Yes	31,007 (80.41)
No or unknown	7552 (19.59)
Nodal radiation	
Yes	26,339 (68.31)
No or unknown	12,220 (31.69)

^a^Facility type is suppressed by the NCDB for patients younger than 40 years

^b^Rurality defined using rural-urban continuum codes (RUCC) where 1–3 is metropolitan and 4–9 is rural

*IQR* interquartile range, *HSD* high school degree, *CoC* Commission on Cancer, *HR* hormone receptor, *SLNB* sentinel lymph node biopsy, *ALND* axillary lymph node dissection

T2 (*n* = 17,784, 46.12%) and N1 (*n* = 30,789, 79.85%) were the most common clinical stages at presentation, and the most common pathologic stage after NAC among this cohort with residual disease on pathology was pN1 (*n* = 23,108, 59.93%). HR+ (*n* = 27,543, 71.43) and HER2− (*n* = 34,226, 88.76%) disease were more common. Most patients underwent mastectomy (*n* = 26,897, 69.76%) compared with partial mastectomy (*n* = 11,311, 29.33%). Mastectomy rates increased with increasing clinical and pathologic N stage (Supplemental Table 1). Additionally, most patients received breast (*n* = 31,007, 80.41%) and nodal (*n* = 26,339, 68.31%) radiation. Additional cohort characteristics are shown in Table 1.

### Unadjusted Bivariate Analyses

For all program types, SLNB alone was the least common axillary surgery type. Integrated Network Programs and Community Programs had the highest rate of SLNB alone (20.19% and 20.15%, respectively) followed by Comprehensive Community Programs (19.33%) and Academic Programs (15.77%) (*p* < 0.01) (Table 2). Integrated Network Programs also had the highest rates of SLNB+ALND (40.35%). For ALND alone, Community and Comprehensive Community Programs had the highest rates (50.34% and 46.81%, respectively).

**Table 2:** Cohort characteristics by type of axillary surgery

Cohort characteristics	SLNB alone (*N* = 7137) *n* (%)	SLNB + ALND (*N* = 14,249) *n* (%)	ALND alone (*N* = 17,173) *n* (%)	*p*
CoC cancer program facility type^a^				< 0.01^c^
Academic	1844 (15.77)	4489 (38.40)	5358 (45.83)	
Comprehensive community	2407 (19.33)	4218 (33.87)	5830 (46.81)	
Integrated network	1493 (20.19)	2984 (40.35)	2919 (39.47)	
Community	413 (20.15)	605 (29.51)	1032 (50.34)	
Unknown	980 (19.73)	1953 (39.32)	2034 (40.95)	
Year of diagnosis				< 0.01^c^
2016	857 (13.96)	1,900 (30.95)	3,381 (55.08)	
2017	881 (14.64)	2,053 (34.11)	3,084 (51.25)	
2018	1067 (16.82)	2143 (33.77)	3135 (49.41)	
2019	1230 (18.59)	2679 (40.48)	2709 (40.93)	
2020	1435 (22.04)	2580 (39.62)	2497 (38.34)	
2021	1667 (24.06)	2894 (41.77)	2367 (34.17)	
Age (years)				< 0.01^d^
Median, IQR	54 (45–63)	54 (45–63)	54 (47–65)	
Sex				< 0.01^c^
Female	7085 (18.54)	14,142 (37.00)	16,990 (44.46)	
Male	52 (15.20)	107 (31.29)	183 (53.51)	
Race				< 0.01^c^
Non-Hispanic White	4697 (19.00)	9291 (37.59)	10,731 (43.41)	
Non-Hispanic Black	1213 (17.12)	2462 (34.74)	3411 (48.14)	
Hispanic	662 (16.87)	1447 (36.88)	1815 (46.25)	
Non-Hispanic Asian	363 (20.40)	678 (38.11)	738 (41.48)	
Non-Hispanic American Indian or Alaskan Native	36 (20.34)	62 (35.03)	79 (44.63)	
Non-Hispanic Native Hawaiian or Pacific Islander	37 (25.52)	39 (26.90)	69 (47.59)	
Other or unknown	129 (17.70)	270 (37.04)	330 (45.27)	
Charlson-Deyo Comorbidity Index				< 0.01^c^
0	6088 (18.83)	12,048 (37.27)	14,193 (43.90)	
1	764 (16.89)	1639 (36.24)	2120 (46.87)	
2	186 (17.75)	363 (34.64)	499 (47.61)	
3+	99 (15.02)	199 (30.20)	361 (54.78)	
Insurance				< 0.01^c^
Private	4359 (19.55)	8833 (39.62)	9101 (40.82)	
Medicare	1614 (17.45)	3057 (33.05)	4579 (49.50)	
Medicaid	819 (16.78)	1622 (33.22)	2441 (50.00)	
Uninsured	161 (14.20)	372 (32.80)	601 (53.00)	
Other government	120 (19.32)	236 (38.00)	265 (42.67)	
Unknown	64 (16.89)	129 (34.04)	186 (49.08)	
Median income				< 0.01^c^
< $46,227	894 (16.12)	1812 (32.68)	2839 (51.20)	
$46,227–$57,856	1183 (17.45)	2345 (34.59)	3252 (47.96)	
$57,857–$74,062	1394 (18.27)	2850 (37.35)	3387 (44.38)	
≥ $74,063	2553 (20.14)	5089 (40.15)	5034 (39.71)	
Unknown	1113 (18.78)	2153 (36.33)	2661 (44.90)	
Education				< 0.01^c^
< 5% with no HSD	1430 (20.23)	2865 (40.52)	2775 (39.25)	
5–9% with no HSD	1773 (18.99)	3546 (37.98)	4017 (43.03)	
9.1–15.2% with no HSD	1588 (17.62)	3264 (36.22)	4160 (46.16)	
≥ 15.3% with no HSD	1246 (17.12)	2440 (33.53)	3591 (49.35)	
Unknown	1100 (18.76)	2134 (36.39)	2630 (44.85)	
Rurality^b^				< 0.01^c^
Metropolitan	6183 (18.73)	12,378 (37.50)	14,446 (43.77)	
Rural	793 (17.26)	1500 (32.65)	2301 (50.09)	
Unknown	161 (16.81)	371 (38.73)	426 (44.47)	
Distance traveled (miles)				< 0.01^c^
< 10	2995 (18.96)	5824 (36.88)	6974 (44.16)	
10–30	2130 (18.98)	4209 (37.51)	4882 (43.51)	
> 30 miles	968 (16.27)	2170 (36.46)	2813 (47.27)	
Unknown	1044 (18.66)	2046 (36.57)	2504 (44.76)	
Clinical T stage				< 0.01^c^
cT1	1386 (22.30)	2594 (41.74)	2235 (35.96)	
cT2	3800 (21.37)	7128 (40.08)	6856 (38.55)	
cT3	1440 (16.41)	3315 (37.77)	4022 (45.82)	
cT4	511 (8.84)	1212 (20.96)	4060 (70.21)	
Clinical N stage				< 0.01^c^
cN1	6281 (20.40)	12,221 (39.69)	12,287 (39.91)	
cN2	485 (11.49)	1175 (27.83)	2562 (60.68)	
cN3	371 (10.46)	853 (24.04)	2324 (65.50)	
Pathologic N stage				
pN1	6181 (26.75)	8707 (37.68)	8220 (35.57)	< 0.01^c^
pN2	809 (7.60)	4065 (38.18)	5774 (54.23)	
pN3	147 (3.06)	1477 (30.75)	3179 (66.19)	
Hormone receptor status				< 0.01^c^
HR+	5194 (18.86)	10,581 (38.42)	11,768 (42.73)	
HR−	1919 (17.62)	3617 (33.21)	5354 (49.16)	
Unknown	24 (19.05)	51 (40.48)	51 (40.48)	
HER2 status				< 0.01^c^
HER2+	729 (20.04)	1207 (33.18)	1702 (46.78)	
HER2−	6287 (18.37)	12,808 (37.42)	15,131 (44.21)	
Unknown	121 (17.41)	234 (33.67)	340 (48.92)	
Breast surgery				< 0.01^c^
Mastectomy	3947 (14.67)	9469 (35.20)	13,481 (50.12)	
Partial mastectomy	3123 (27.61)	4640 (41.02)	3548 (31.37)	
Unknown	67 (19.09)	140 (39.89)	144 (41.03)	
Breast radiation				< 0.01^c^
Yes	5813 (18.75)	11,758 (37.92)	13,436 (43.33)	
No or unknown	1324 (17.53)	2491 (32.98)	3737 (49.48)	
Nodal radiation				< 0.01^c^
Yes	4829 (18.33)	10,095 (38.33)	11,415 (43.34)	
No or unknown	2308 (18.89)	4154 (33.99)	5758 (47.12)	

^a^Facility type is suppressed by the NCDB for patients younger than 40 years

^b^Rurality defined using rural-urban continuum codes (RUCC) where 1–3 is metropolitan and 4–9 is rural

^c^*p*-values obtained from Chi-square test

^d^*p*-values obtained from Kruskal-Wallis test

*IQR* interquartile range, *HSD* high school degree, *CoC* Commission on Cancer, *HR* hormone receptor, *SLNB* sentinel lymph node biopsy, *ALND* axillary lymph node dissection

**Fig. 2:**
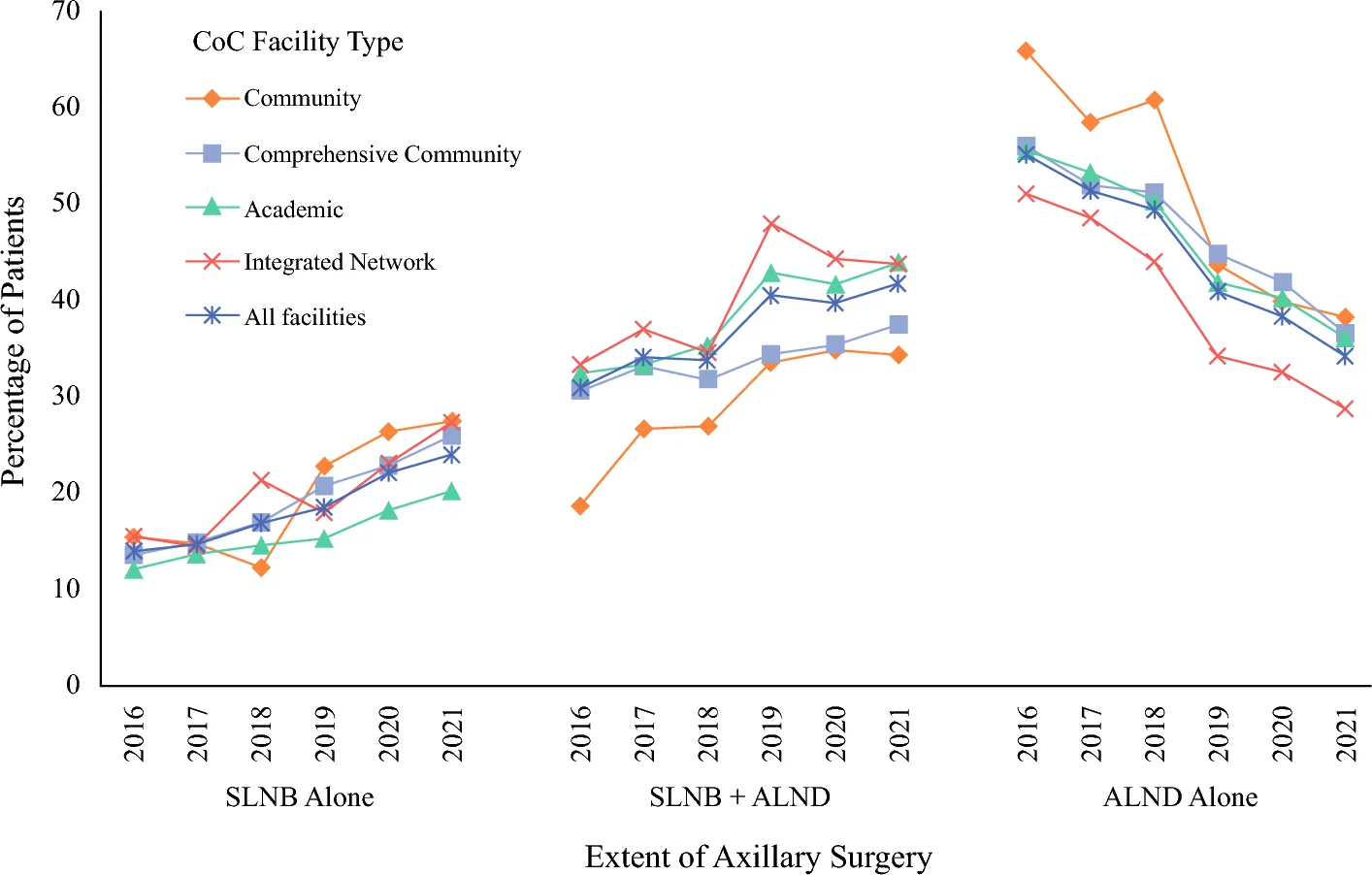
Percentage of patients receiving each category of axillary management across Commission on Cancer facility types over time

The percentage of patients receiving SLNB alone and SLNB+ALND increased over the evaluated time period across all facility types, whereas the percentage of patients receiving ALND alone decreased across all facility types (Fig. 2; Table 2). Additional cohort details by axillary surgery type are shown in Table 2.

### Adjusted Multivariable Analyses

In the analysis restricted to patients who underwent initial SLNB, compared with academic programs, patients treated at comprehensive community, integrated network, community, and unknown program type had greater odds of SLNB alone compared with SLNB+ALND (odds ratio [OR] 1.32, 95% confidence interval [CI] 1.22–1.43; OR 1.17, 95% CI 1.07–1.27; OR 1.54, 95% CI 1.33–1.78; OR 1.17, 95% CI 1.03–1.33, respectively). This remained true for patients with pN1 disease at all facility types and for patients who had higher (i.e., pN2–pN3) nodal disease burden treated at community and comprehensive community programs (Supplemental Tables 2, 3).

**Table 3:** Multivariable logistic regression model demonstrating factors associated with SLNB alone (reference is SLNB + ALND) for breast cancer patients (*N* = 21,386) with pN+ disease after neoadjuvant chemotherapy adjusting for covariates

Cohort characteristics	OR (95% CI)	*p*
CoC cancer program facility type^a^		
Academic	*Reference*	
Comprehensive community	1.32 (1.22, 1.43)	< 0.01
Integrated network	1.17 (1.07, 1.27)	< 0.01
Community	1.54 (1.33, 1.78)	< 0.01
Unknown	1.17 (1.03, 1.33)	0.01
Year of diagnosis	1.07 (1.05, 1.09)	< 0.01
Age (years)	1.00 (0.99, 1.00)	0.32
Sex		
Female	*Reference*	
Male	0.98 (0.69, 1.38)	0.89
Race		
Non-Hispanic White	*Reference*	
Non-Hispanic Black	0.95 (0.88, 1.04)	0.29
Hispanic	0.90 (0.80, 1.00)	0.05
Non-Hispanic Asian	0.99 (0.86, 1.13)	0.85
Non-Hispanic American Indian or Alaskan Native	1.12 (0.73, 1.73)	0.61
Non-Hispanic Native Hawaiian or Pacific Islander	1.77 (1.10, 2.83)	0.02
Other or unknown	0.94 (0.75, 1.18)	0.60
Charlson-Deyo Comorbidity Index		
0	*Reference*	
1	0.90 (0.82, 0.99)	0.04
2	1.05 (0.87, 1.27)	0.61
3+	0.90 (0.69, 1.16)	0.40
Insurance		
Private	*Reference*	
Medicare	1.09 (0.99, 1.20)	0.08
Medicaid	1.06 (0.96, 1.17)	0.22
Uninsured	0.90 (0.74, 1.10)	0.30
Other government	1.07 (0.84, 1.35)	0.59
Unknown	1.10 (0.80, 1.51)	0.57
Median income		
< $46,227	*Reference*	
$46,227–$57,856	1.03 (0.92, 1.16)	0.63
$57,857–$74,062	1.00 (0.89, 1.13)	0.95
≥ $74,063	1.02 (0.90, 1.16)	0.72
Unknown	1.33 (0.63, 2.80)	0.45
Education		
< 5% with no HSD	*Reference*	
5–9% with no HSD	0.98 (0.89, 1.07)	0.63
9.1–15.2% with no HSD	0.98 (0.88, 1.08)	0.65
≥ 15.3% with no HSD	1.03 (0.91, 1.16)	0.66
Unknown	0.96 (0.42, 2.17)	0.92
Rurality^b^		
Metropolitan	*Reference*	
Rural	1.05 (0.94, 1.17)	0.39
Unknown	0.97 (0.80, 1.19)	0.81
Distance traveled (miles)		
< 10	*Reference*	
10–30	1.01 (0.94, 1.09)	0.81
> 30	0.90 (0.81, 1.00)	0.05
Unknown	0.80 (0.55, 1.14)	0.22
Clinical T stage		
cT1	*Reference*	
cT2	1.05 (0.97, 1.13)	0.27
cT3	1.07 (0.97, 1.18)	0.16
cT4	1.13 (0.99, 1.29)	0.07
Clinical N stage		
cN1	*Reference*	
cN2	1.02 (0.90, 1.14)	0.79
cN3	1.11 (0.97, 1.27)	0.14
Pathologic N stage		
pN1	*Reference*	
pN2	0.29 (0.27, 0.31)	< 0.01
pN3	0.15 (0.12, 0.17)	< 0.01
Hormone receptor status		
HR+	*Reference*	
H−	0.97 (0.90, 1.04)	0.33
Unknown	0.86 (0.50, 1.49)	0.59
HER2 status		
HER2+	*Reference*	
HER2−	0.92 (0.83, 1.02)	0.10
Unknown	0.99 (0.76, 1.30)	0.96
Breast surgery		
Mastectomy	*Reference*	
Partial mastectomy	1.46 (1.37, 1.56)	< 0.01
Unknown	1.15 (0.85, 1.56)	0.36
Breast radiation		
No or unknown	*Reference*	
Yes	0.98 (0.89, 1.08)	0.64
Nodal radiation		
No or unknown	*Reference*	
Yes	0.87 (0.81, 0.95)	< 0.01

^a^Facility type is suppressed by the NCDB for patients younger than 40 years

^b^Rurality defined using rural-urban continuum codes (RUCC) where 1–3 is metropolitan and 4–9 is rural

*HSD* high school degree, *CoC* Commission on Cancer, *HR* hormone receptor, *SLNB* sentinel lymph node biopsy, *ALND* axillary lymph node dissection, *OR* odds ratio, *CI* confidence interval

With each passing year since 2016, patients had greater odds of undergoing SLNB alone compared to SLNB+ALND (OR 1.07, 95% CI 1.05–1.09) (Table 3). There was no difference in axillary surgery by age or sex. Race and ethnicity also were not associated with axillary surgery except for patients who are Non-Hispanic Native Hawaiian or Pacific Islander who had greater odds of SLNB alone compared to non-Hispanic White patients (OR 1.77, 95% CI 1.1–2.83). Charlson–Deyo Comorbidity Index of 1 was associated with decreased odds of SLNB alone compared to Index of 0 (OR 0.90, 95% CI 0.82–0.99), but there was no association between axillary surgery and higher indices i.e. Charlson–Deyo Comorbidity Index ≥ 2. For socioeconomic factors, there was no association between axillary surgery and insurance status, median household income, or education. Similarly, there was no difference between axillary surgery and rurality or distance traveled.

Several clinical characteristics were associated with axillary surgery type, including pN stage, type of breast surgery, and receipt of nodal radiation. Pathologic N stage had the strongest association with axillary surgery type; patients with pN2 and pN3 disease had significantly lower odds of SLNB alone than SLNB+ALND (OR 0.29, 95% CI 0.27–0.31 and OR 0.15, 95% CI 0.17 for pN2 and pN3, respectively). There was no association between axillary surgery and cT stage or cN stage. Patients who underwent a partial mastectomy had greater odds of SLNB alone compared with those who underwent mastectomy (OR 1.46, 95% CI 1.37–1.56). Those who underwent nodal radiation had lower odds of SLNB alone than SLNB + ALND (OR 0.87, 95% CI 0.81–0.95). There was no association between HR receptor status, HER2 status, or breast radiation. Additional results for the multivariable logistic regression model demonstrating factors associated with SLNB alone in patients with pN+ disease are shown in Table 3.

## Discussion

This retrospective cohort study demonstrated facility-level variation in de-escalation of axillary surgery for patients with clinically involved regional nodes who received NAC and had residual axillary involvement, with greatest omission of ALND observed in community facility types. There was overall increased omission of ALND at all facility types over time. Furthermore, facility type effects varied by extent of residual nodal disease.

Care at an accredited cancer center is associated with improved oncologic outcomes through care standardization and quality metrics.^22–25^ However, prior work has demonstrated variation in adoption of evidence by facility type. For example, a study evaluating compliance among CoC facilities after introduction of the CoC’s quality measure requiring resection and examination of at least 12 regional lymph nodes during surgery for colon cancer found that while all facility types improved, facilities with higher accreditation levels (e.g., NCI designated and Academic Programs) had greater compliance and in risk-adjusted models had better survival.^26^ Similarly, another study evaluated de-implementation of completion lymph node dissection (CLND) in melanoma patients after results of MSLT-II and DeCOG-SLT, which provided evidence to support the use of surveillance in patients within nodal disease. They found that patients treated at academic programs had higher rates of CLND compared with community and integrated network programs.^18^ Additionally, surgery at a community cancer program was associated with decreased odds of CLND compared with an academic program amongst patients who underwent systemic therapy. This was consistent with our findings that community cancer programs were more likely to de-escalate nodal surgery; however, de-implementation of CLND for melanoma was based on concordant results from two large, randomized clinical trials compared with de-escalation of ALND for breast cancer patients with residual nodal disease on pathology, which remains an area of active investigation. Another study that evaluated oncologic outcomes after pancreatectomy demonstrated improved disease-specific survival and overall survival at NCI-designated sites compared with CoC-accredited and non-accredited sites and no difference in these outcomes between CoC-accredited and non-accredited sites.^27^ Similarly, metropolitan healthcare markets with an NCI-designated cancer center were associated with lower population cancer mortality rates than those without a cancer center. There was a similar directional effect for areas with an Academic or Comprehensive Community Program, but this did not reach significance.^28^ Further work to elucidate factors contributing to these observed facility‑level differences and strategies to ensure all cancer facilities appropriately apply evidence-based care will be critical to promoting equitable access to high-quality care.

This study also demonstrated a temporal trend in patients with cN+ patients found to be ypN+. Rates of ALND decreased across all facility types over time, and this pattern is similar to results seen in other studies.^29,30^ Data from patients enrolled in the I-SPY2 trial found that in patients who were ypN+, the rate of ALND alone decreased from 69.0 to 39.2%, whereas SLNB alone rose from 6.9 to 39.2% between 2011 and 2021.^31^ This study also uniquely showed that in adjusted analysis, the odds of omission of ALND after initial SLNB in patients with residual pathologic disease increased over time. The decline in initial ALND alone and the concurrent rise in both SLNB alone and SLNB+ALND over time, along with increasing odds of ALND omission after initial SLNB over time in adjusted analyses, may suggest that guidelines from previous studies supporting omission of ALND in select populations are being applied to populations to which the data does not yet apply or is currently under investigation. Similar effects have been seen in breast cancer care previously. For example, after the U.S. Preventative Services Task Force (USPSTF) changed recommendations for breast cancer screening allowing for less stringent screening in younger women, it was found that older women who were not included in these recommendations experienced declines in screening as well.^20^ Similarly, after Z0011 where there was a degree of de-escalation of ALND in women with three to four positive nodes that were not included in the Z0011 trial.^17^ Alternatively, it is possible that providers may be anticipating results of ongoing clinical trials investigating omission of ALND in select populations with residual nodal disease after NAC.

Another factor that was associated with SLNB alone compared with SLNB+ALND was partial mastectomy. This relationship was stronger in patients who had pN1 disease compared with pN2-pN3 disease. Additionally, with increasing pathologic nodal burden, the percentage of patients who underwent mastectomy increased, although this is not a known factor when the decision to perform a partial mastectomy versus mastectomy is made. This pattern was similarly seen with increasing clinical nodal burden. While the decision to perform a partial mastectomy versus a mastectomy is nuanced and balances both clinical factors and patient preference, this suggests that clinical nodal burden may influence decision-making regarding pursuing breast conserving therapy. Alternatively, those who had greater extent of disease may not have responded as well to NAC, whereas patients who received breast conservation may have had a more favorable response to NAC and thus were more likely to have SLNB alone. Conversely, nodal radiation was associated with lower odds of SLNB alone. Current NCCN guidelines recommend regional nodal radiation in patients ypN+ after NAC.^16^ It is possible that patients for whom ALND is omitted are deemed overall lower-risk by their providers and thus not requiring nodal radiation. While some trials are evaluating the role of nodal radiation with or without ALND in patients who are ypN+ after NAC, there are currently no indications for nor trials evaluating omission of nodal radiation and ALND in this population.

Higher pathologic nodal stages, i.e., pN2 and pN3 were significantly associated with decreased odds of SLNB alone compared with SLNB + ALND. While this pattern suggests that greater nodal burden, not just the presence of nodal disease on surgery, influences whether surgeons proceed to ALND, it also highlights the broader clinical debate regarding whether all patients with residual nodal disease after NAC, in particular those with pN1 involvement, require ALND. At present, in patients with residual axillary disease after NAC, ALND remains the standard of care,^15,16^ and prior work has demonstrated that greater than 60% of nonsentinel nodes are positive in patients with ypN+ disease.^32^ However, there is a growing pool of evidence to suggest that patients who are pathologically N1 after NAC may not require ALND, particularly when adjuvant therapy is planned. In studies where patients who had residual pN1 disease after NAC, recurrence rates as well as disease-free survival and overall survival were similar amongst those who underwent SLNB alone and SLNB+ALND.^33,34^ There is also data to support a low axillary recurrence rate in patients with pN+ disease after NAC where ALND was omitted.^35^ Furthermore, an international, multicenter study found that among patients with micrometastases after NAC, there was no significant difference in axillary recurrence amongst those who underwent ALND, although there were differences specifically for patients with triple-negative disease.^36^ Additionally, data from the I-SPY2 trial demonstrated that in patients with ypN+ breast cancer, there was no significant difference in 5-year rates of axillary or locoregional recurrence, distant recurrence-free survival or event-free survival.^37^ Together, these studies suggest that omission of ALND, while not currently standard of care in this population, may be appropriate in select patients with residual nodal disease after NAC pending the results of ongoing clinical trials expected to further inform this practice.

At present, multiple clinical trials are underway to evaluate axillary management strategies in patients with residual disease after NAC.^38^ The Alliance A011202 clinical trial compares ALND with regional nodal radiation to axillary and regional nodal radiation without ALND among patients with positive sentinel lymph node disease after NAC.^39^ Among patients who had a positive sentinel lymph node biopsy after neoadjuvant systemic therapy (chemotherapy or endocrine therapy), ADARNAT compares ALND alone to axillary radiation therapy alone.^40^ TAXIS is a clinical trial comparing tailored axillary surgery followed by ALND and regional nodal irradiation (excluding the dissected axilla) to tailored axillary surgery followed by regional nodal irradiation (including the full axilla) among patients with positive nodal disease in the adjuvant setting or residual nodal disease after NAC.^41^ The findings of these trials will help to guide future recommendations for axillary management in patients with cN+ disease who remain node-positive after NAC.

This study is subject to limitations including those inherent to the use of retrospective tumor registry data from participating CoC facilities. The NCDB does not include patients who are treated outside accredited CoC facilities. Facility type is suppressed for patients younger than 40 years, limiting generalizability of the results about facility types to younger patients. Finally, clinical stage after NAC was not known, and post-NAC staging was inferred from pathologic stage. This is particularly important limitation as decision on surgery is usually made based on clinical nodal status after NAC, which is important when thinking about practice patterns. Additionally, the surgical decisions as result of multi-disciplinary discussion is also unknown.

## Conclusions

This study evaluated factors associated with omission of ALND in patients with residual nodal disease after NAC undergoing upfront SLNB. While ALND has been de-escalated with a concurrent rise in SLNB alone in this population despite ongoing evaluation of this approach, the odds of omission of ALND were greatest at non-Academic Cancer Centers. The Alliance A011202, ADARNAT, and TAXIS clinical trials will provide additional insight on how to treat the axilla for patients with residual axillary disease after NAC. Until then, current evidence should be weighed in a multidisciplinary discussion along with patient-provider shared decision making to determine the best treatment course for the patient and ensure evidence-based recommendations are appropriately applied.

## Supplementary Information

Below is the link to the electronic supplementary material.

Supplementary file1 (DOCX 50 kb)
