# Supplementary material for: Factors Associated with Omission of Axillary Lymph Node Dissection for Patients with Clinical Node-Positive Breast Cancer Who have Residual Nodal Disease on Pathology After Neoadjuvant Chemotherapy
Source: Ann Surg Oncol. 2026 Jun 26;33(10):9035–48. doi: 10.1245/s10434-026-19968-5 (PMC13585772; doi:10.1245/s10434-026-19968-5)
Supplement: Supplementary file 1 [file 10434_2026_19968_MOESM1_ESM.docx]

**Supplemental Table 1.** Breast surgery stratified by clinical and pathologic nodal stage.

| **Breast surgery** | **Clinical nodal stage (n, %)** | | | **Pathologic nodal stage (n,%)** | | |
| --- | --- | --- | --- | --- | --- | --- |
|  | **cN1** | **cN2** | **cN3** | **pN1** | **pN2** | **pN3** |
| Mastectomy | 20,852 (67.73) | 3,181 (75.34) | 2,864 (80.72) | 14,781 (63.96) | 8,143 (76.47) | 3,973 (82.72) |
| Partial mastectomy | 9,659 (31.37) | 1,007 (23.85) | 645 (18.18) | 8,126 (35.17) | 2,397 (22.51) | 788 (16.41) |
| Unknown | 278 (0.90) | 34 (0.81) | 39 (1.10) | 201 (0.87) | 108 (1.01) | 42 (0.87) |

**Supplemental Table 2.** Multivariable logistic regression model demonstrating factors associated with SLNB alone (reference is SLNB + ALND) for breast cancer patients (N=14,888) with pN1 disease after neoadjuvant chemotherapy adjusting for covariates.

| **Cohort characteristics** | **OR (95% CI)** | **P-Value** |
| --- | --- | --- |
| **CoC Cancer Program Facility Type^1^** |  |  |
| Academic | *Reference* |  |
| Comprehensive Community | 1.36 (1.24, 1.48) | <0.01 |
| Integrated Network | 1.19 (1.08, 1.31) | <0.01 |
| Community | 1.53 (1.30, 1.81) | <0.01 |
| Unknown | 1.18 (1.03, 1.35) | 0.02 |
| **Year of Diagnosis** | 1.05 (1.03, 1.08) | <0.01 |
| **Age (years)** | 1.00 (0.99, 1.00) | 0.18 |
| **Sex** |  |  |
| Female | *Reference* |  |
| Male | 0.94 (0.64, 1.37) | 0.74 |
| **Race** |  |  |
| Non-Hispanic White | *Reference* |  |
| Non-Hispanic Black | 0.95 (0.86, 1.04) | 0.25 |
| Hispanic | 0.90 (0.80, 1.02) | 0.09 |
| Non-Hispanic Asian | 0.98 (0.84, 1.14) | 0.78 |
| Non-Hispanic American Indian or Alaskan Native | 1.03 (0.64, 1.67) | 0.90 |
| Non-Hispanic Native Hawaiian or Pacific Islander | 1.44 (0.85, 2.43) | 0.18 |
| Other or unknown | 0.93 (0.73, 1.19) | 0.56 |
| **Charlson-Deyo Comorbidity Index** |  |  |
| 0 | *Reference* |  |
| 1 | 0.91 (0.82, 1.01) | 0.08 |
| 2 | 1.05 (0.84, 1.31) | 0.67 |
| 3+ | 0.82 (0.61, 1.09) | 0.17 |
| **Insurance** |  |  |
| Private | *Reference* |  |
| Medicare | 1.13 (1.02, 1.26) | 0.03 |
| Medicaid | 1.06 (0.95, 1.18) | 0.30 |
| Uninsured | 0.90 (0.72, 1.12) | 0.34 |
| Other Government | 1.09 (0.84, 1.41) | 0.51 |
| Unknown | 1.24 (0.87, 1.77) | 0.23 |
| **Median Income** |  |  |
| <$46,227 | *Reference* |  |
| $46,227-$57,856 | 1.05 (0.93, 1.20) | 0.43 |
| $57,857-$74,062 | 0.99 (0.87, 1.14) | 0.92 |
| ≥$74,063 | 1.06 (0.92, 1.22) | 0.43 |
| Unknown | 1.75 (0.74, 4.11) | 0.20 |
| **Education** |  |  |
| <5% with no HSD | *Reference* |  |
| 5.0%-9.0% with no HSD | 0.98 (0.89, 1.09) | 0.75 |
| 9.1%-15.2% with no HSD | 1.00 (0.89, 1.12) | 0.94 |
| ≥15.3% with no HSD | 1.04 (0.90, 1.19) | 0.61 |
| Unknown | 0.87 (0.34, 2.20) | 0.76 |
| **Rurality^2^** |  |  |
| Metropolitan | *Reference* |  |
| Rural | 1.11 (0.98, 1.25) | 0.10 |
| Unknown | 1.04 (0.83, 1.30) | 0.73 |
| **Distance traveled** |  |  |
| <10 miles | *Reference* |  |
| 10-30 miles | 0.99 (0.91, 1.07) | 0.72 |
| >30 miles | 0.88 (0.78, 0.99) | 0.03 |
| Unknown | 0.70 (0.47, 1.04) | 0.07 |
| **Clinical T Stage** |  |  |
| cT1 | *Reference* |  |
| cT2 | 1.01 (0.93, 1.10) | 0.79 |
| cT3 | 1.04 (0.94, 1.16) | 0.45 |
| cT4 | 1.02 (0.88, 1.19) | 0.79 |
| **Clinical N stage** |  |  |
| cN1 | *Reference* |  |
| cN2 | 0.91 (0.79, 1.05) | 0.21 |
| cN3 | 0.98 (0.83, 1.15) | 0.82 |
| **Hormone Receptor Status** |  |  |
| HR+ | *Reference* |  |
| HR- | 0.95 (0.88, 1.03) | 0.22 |
| Unknown | 0.90 (0.50, 1.63) | 0.74 |
| **HER2 Status** |  |  |
| HER2+ | *Reference* |  |
| HER2- | 0.88 (0.79, 0.99) | 0.03 |
| Unknown | 0.99 (0.74, 1.33) | 0.96 |
| **Breast Surgery** |  |  |
| Mastectomy | *Reference* |  |
| Partial Mastectomy | 1.49 (1.38, 1.60) | <0.01 |
| Unknown | 1.10 (0.78, 1.55) | 0.58 |
| **Breast Radiation** |  |  |
| No or unknown | *Reference* |  |
| Yes | 0.99 (0.89, 1.10) | 0.86 |
| **Nodal Radiation** |  |  |
| No or unknown | *Reference* |  |
| Yes | 0.94 (0.86, 1.03) | 0.19 |

^1^Note: Facility type is suppressed by the NCDB for patients younger than 40 years.
^2^Rurality defined using rural-urban continuum codes (RUCC) where 1-3 is metropolitan and 4-9 is rural
HSD= high school degree, CoC= Commission on Cancer, HR= hormone receptor, SLNB= sentinel lymph node biopsy, ALND= axillary lymph node dissection

**Supplemental Table 3.** Multivariable logistic regression model demonstrating factors associated with SLNB alone (reference is SLNB + ALND) for breast cancer patients (N=4,146) with pN2 or pN3 disease after neoadjuvant chemotherapy adjusting for covariates.

| **Cohort characteristics** | **OR (95% CI)** | **P-Value** |
| --- | --- | --- |
| **CoC Cancer Program Facility Type^1^** |  |  |
| Academic | *Reference* |  |
| Comprehensive Community | 1.23 (1.03, 1.48) | 0.02 |
| Integrated Network | 1.12 (0.92, 1.38) | 0.26 |
| Community | 1.48 (1.07, 2.05) | 0.02 |
| Unknown | 1.15 (0.85, 1.56) | 0.36 |
| **Year of Diagnosis** | 1.14 (1.10, 1.19) | <0.01 |
| **Age (years)** | 1.00 (0.99, 1.01) | 0.64 |
| **Sex** |  |  |
| Female | *Reference* |  |
| Male | 1.36 (0.62, 2.99) | 0.45 |
| **Race** |  |  |
| Non-Hispanic White | *Reference* |  |
| Non-Hispanic Black | 0.95 (0.78, 1.16) | 0.62 |
| Hispanic | 0.88 (0.68, 1.14) | 0.33 |
| Non-Hispanic Asian | 1.05 (0.74, 1.50) | 0.78 |
| Non-Hispanic American Indian or Alaskan Native | 1.45 (0.57, 3.69) | 0.43 |
| Non-Hispanic Native Hawaiian or Pacific Islander | 4.17 (1.63, 10.67) | <0.01 |
| Other or unknown | 0.95 (0.56, 1.58) | 0.83 |
| **Charlson-Deyo Comorbidity Index** |  |  |
| 0 | *Reference* |  |
| 1 | 0.85 (0.67, 1.07) | 0.16 |
| 2 | 1.08 (0.73, 1.59) | 0.69 |
| 3+ | 1.29 (0.74, 2.22) | 0.37 |
| **Insurance** |  |  |
| Private | *Reference* |  |
| Medicare | 0.88 (0.70, 1.10) | 0.26 |
| Medicaid | 1.06 (0.85, 1.31) | 0.63 |
| Uninsured | 0.86 (0.53, 1.40) | 0.55 |
| Other Government | 1.02 (0.58, 1.80) | 0.93 |
| Unknown | 0.65 (0.27, 1.52) | 0.32 |
| **Median Income** |  |  |
| <$46,227 | *Reference* |  |
| $46,227-$57,856 | 0.90 (0.69, 1.19) | 0.46 |
| $57,857-$74,062 | 1.01 (0.77, 1.33) | 0.95 |
| ≥$74,063 | 0.85 (0.64, 1.14) | 0.29 |
| Unknown | 0.49 (0.06, 4.02) | 0.51 |
| **Education** |  |  |
| <5% with no HSD | *Reference* |  |
| 5.0%-9.0% with no HSD | 0.94 (0.75, 1.17) | 0.58 |
| 9.1%-15.2% with no HSD | 0.91 (0.71, 1.16) | 0.43 |
| ≥15.3% with no HSD | 1.01 (0.76, 1.34) | 0.96 |
| Unknown | 0.92 (0.09, 9.88) | 0.95 |
| **Rurality^2^** |  |  |
| Metropolitan | *Reference* |  |
| Rural | 0.82 (0.63, 1.07) | 0.14 |
| Unknown | 0.74 (0.44, 1.23) | 0.25 |
| **Distance traveled** |  |  |
| <10 miles | *Reference* |  |
| 10-30 miles | 1.13 (0.95, 1.34) | 0.16 |
| >30 miles | 1.00 (0.78, 1.27) | 0.97 |
| Unknown | 1.84 (0.58, 5.79) | 0.30 |
| **Clinical T Stage** |  |  |
| cT1 | *Reference* |  |
| cT2 | 1.29 (1.04, 1.61) | 0.02 |
| cT3 | 1.24 (0.98, 1.58) | 0.08 |
| cT4 | 1.51 (1.14, 2.00) | <0.01 |
| **Clinical N stage** |  |  |
| cN1 | *Reference* |  |
| cN2 | 1.31 (1.07, 1.61) | 0.01 |
| cN3 | 1.35 (1.07, 1.71) | 0.01 |
| **Hormone Receptor Status** |  |  |
| HR+ | *Reference* |  |
| HR- | 1.00 (0.84, 1.19) | 0.96 |
| Unknown | 0.57 (0.11, 2.84) | 0.49 |
| **HER2 Status** |  |  |
| HER2+ | *Reference* |  |
| HER2- | 1.14 (0.85, 1.53) | 0.39 |
| Unknown | 1.14 (0.56, 2.28) | 0.72 |
| **Breast Surgery** |  |  |
| Mastectomy | *Reference* |  |
| Partial Mastectomy | 1.34 (1.13, 1.58) | <0.01 |
| Unknown | 1.31 (0.69, 2.49) | 0.41 |
| **Breast Radiation** |  |  |
| No or unknown | *Reference* |  |
| Yes | 0.94 (0.76, 1.16) | 0.55 |
| **Nodal Radiation** |  |  |
| No or unknown | *Reference* |  |
| Yes | 0.64 (0.54, 0.77) | <0.01 |

^1^Note: Facility type is suppressed by the NCDB for patients younger than 40 years.
^2^Rurality defined using rural-urban continuum codes (RUCC) where 1-3 is metropolitan and 4-9 is rural
HSD= high school degree, CoC= Commission on Cancer, HR= hormone receptor, SLNB= sentinel lymph node biopsy, ALND= axillary lymph node dissection

**STROBE Checklist**

|  | Item No. | Recommendation | Page  No. | Relevant text from manuscript |
| --- | --- | --- | --- | --- |
| **Title and abstract** | 1 | (*a*) Indicate the study’s design with a commonly used term in the title or the abstract | 4 | Retrospective observational study |
|  |  | (*b*) Provide in the abstract an informative and balanced summary of what was done and what was found | 4 | *See abstract* |
| Introduction | | | |  |
| Background/rationale | 2 | Explain the scientific background and rationale for the investigation being reported | 5 | For clinically node positive (cN+) patients who receive NAC, historical surgical standard of care has been ALND. However, recent studies have shown that in select patients with cN+ breast cancer who convert to clinically node negative (cN0) after NAC, sentinel lymph node biopsy (SLNB) or SLNB with removal of the marked/clipped node (targeted axillary dissection [TAD]) are appropriate and can be used for staging and definitive axillary surgery for those achieving ypN0. Currently, for patients with cN+ disease who have residual axillary disease after NAC, ALND remains the standard of care.  When a high degree of de-implementation occurs, as was evidenced by the change in ALND rates after the dissemination of the Z0011 trial results, practices such as ALND may be omitted in populations for which it has not yet been studied or remains an active area of ongoing research… Understanding the unintended consequences of de-implementation, in particular the extrapolation of evidence to populations for which the practice has not been thoroughly studied, and which practice locations are at risk, is critical to helping assure evidence-based care is appropriately applied. |
| Objectives | 3 | State specific objectives, including any prespecified hypotheses | 6 | In this study, we sought to evaluate factors associated with omission of ALND after SLNB among patients with residual nodal disease after neoadjuvant chemotherapy. |
| Methods | | | |  |
| Study design | 4 | Present key elements of study design early in the paper | 6 | This was a retrospective observational study using 2016-2021 National Cancer Database (NCDB) data to evaluate factors associated with omission of ALND after initial SLNB for cN+ patients found to be ypN+. |
| Setting | 5 | Describe the setting, locations, and relevant dates, including periods of recruitment, exposure, follow-up, and data collection | 6 | The NCDB Participant User File for breast cancer was queried for patients formally diagnosed between 2016 and 2021 who had part or all of their treatment done at the reporting facility. This period was chosen because it follows publication of SENTINA, ACOSOG Z1071, and SN FNAC, all of which may have contributed to de-escalation of ALND after NAC, and because it was the most recent data available at the time of analysis. The cohort was restricted to patients <90 years old with clinically staged T1-T4 (cT1-cT4), N1-N3 (cN1-cN3) breast cancer who received NAC and had residual nodal disease (pathologic node positive) on axillary surgery (ypN+) (Figure 1). Patients with isolated tumor cells (itc+) or were molecularly positive (mol+) were classified pathologically node negative (pN0) and thus excluded. |
| Participants | 6 | (*a*) *Cohort study*—Give the eligibility criteria, and the sources and methods of selection of participants. Describe methods of follow-up  *Case-control study*—Give the eligibility criteria, and the sources and methods of case ascertainment and control selection. Give the rationale for the choice of cases and controls  *Cross-sectional study*—Give the eligibility criteria, and the sources and methods of selection of participants | 6 | *See above* |
|  |  | (*b*) *Cohort study*—For matched studies, give matching criteria and number of exposed and unexposed  *Case-control study*—For matched studies, give matching criteria and the number of controls per case | N/A |  |
| Variables | 7 | Clearly define all outcomes, exposures, predictors, potential confounders, and effect modifiers. Give diagnostic criteria, if applicable | 6-7 | Primary Outcome and Exposure  The primary outcome was type of axillary surgery. This was categorized into three groups: SLNB alone, SLNB followed by ALND (SLNB+ALND), and ALND alone. The primary exposure of interest was CoC facility type (Academic/Research Program, Comprehensive Community Cancer Program, Community Cancer Program, Integrated Network Cancer Program).  Secondary Exposures  Patient sociodemographic characteristics included age, sex, race and ethnicity, Charlson-Deyo comorbidity score, and insurance status. Area level, zip-code based variables included education, defined as percentage with a high school degree (<5.0%, 5.0%-9.0%, 9.1%-15.2%, ≥15.3%+), and median household income (<$46,277, $46,277-$57,856, $57,857-$74,062, and ≥$74,063) which were categorized into quartiles within the NCDB. Geographic characteristics included distance traveled and rurality. Distance traveled was defined as crowfly distance and was categorized into three groups: <10 miles, 10-30 miles, and > 30 miles. Rurality was defined using the Rural-Urban Continuum Code (RUCC) with 1-3 classified as metropolitan and 4-9 classified as rural. Breast cancer disease and treatment characteristics included clinical T and clinical N stage, pathologic N stage, hormone receptor (HR) status, human epidermal growth factor-2 (HER2) receptor status, type of breast surgery, breast radiation, and nodal radiation. Breast surgery was categorized as mastectomy or partial mastectomy. Year of diagnosis was treated as a continuous variable in regression models (2016-2021). |
| Data sources/ measurement | 8* | For each variable of interest, give sources of data and details of methods of assessment (measurement). Describe comparability of assessment methods if there is more than one group | 6-7 | *See above* |
| Bias | 9 | Describe any efforts to address potential sources of bias | 7-8 | A multivariable logistic regression model was created to evaluate the odds of omission of ALND after receiving SLNB alone and was adjusted for sociodemographic, geographic, and clinical characteristics. Additional subgroup analyses were performed by pN stage (pN1, pN2 and pN3) to examine whether time and facility type effects were consistent across degree of pathologic nodal involvement and to identify influential factors within pathologic nodal stage groups |
| Study size | 10 | Explain how the study size was arrived at | 6, Fig 1 | The NCDB Participant User File for breast cancer was queried for patients formally diagnosed between 2016 and 2021 who had part or all of their treatment done at the reporting facility. The cohort was restricted to patients <90 years old with clinically staged T1-T4 (cT1-cT4), N1-N3 (cN1-cN3) breast cancer who received NAC and had residual nodal disease (pathologic node positive) on axillary surgery (ypN+) (Figure 1). |

| Quantitative variables | 11 | Explain how quantitative variables were handled in the analyses. If applicable, describe which groupings were chosen and why | 6-7 | *See above* |
| --- | --- | --- | --- | --- |
| Statistical methods | 12 | (*a*) Describe all statistical methods, including those used to control for confounding | 7-8 | Descriptive statistics were calculated. Categorical variables are described as frequencies with percentages. Bivariate analyses were performed to evaluate cohort characteristics by axillary surgery type using a chi-square test or Kruskal-Wallis test as appropriate. A multivariable logistic regression model was created to evaluate the odds of omission of ALND after receiving SLNB alone and was adjusted for sociodemographic, geographic, and clinical characteristics. Additional subgroup analyses were performed by pN stage (pN1, pN2 and pN3) to examine whether time and facility type effects were consistent across degree of pathologic nodal involvement and to identify influential factors within pathologic nodal stage groups. Observations with missing or unknown values were retained as a separate category and included in models as such. Results are reported as odds ratios (OR) with 95% confidence intervals (95% CI). Statistical analyses were conducted in Stata Version 18.0 (StataCorp, College Station, TX). |
|  |  | (*b*) Describe any methods used to examine subgroups and interactions | 7-8 | *See above* |
|  |  | (*c*) Explain how missing data were addressed | 8 Fig 1 | Observations with missing or unknown values were retained as a separate category and included in models as such. Figure 1 |
|  |  | (*d*) *Cohort study*—If applicable, explain how loss to follow-up was addressed  *Case-control study*—If applicable, explain how matching of cases and controls was addressed  *Cross-sectional study*—If applicable, describe analytical methods taking account of sampling strategy | N/A |  |
|  |  | (*e*) Describe any sensitivity analyses | N/A |  |
| Results | | | | |
| Participants | 13* | (a) Report numbers of individuals at each stage of study—eg numbers potentially eligible, examined for eligibility, confirmed eligible, included in the study, completing follow-up, and analysed | 8 | A total of 1,508,483 cases of breast cancer diagnosed between 2016-2021 among patients age 18 to 89 (Figure 1) were identified. Patients with all treatment decisions made elsewhere (n=67,206) were excluded. An additional 1,250,887 patients were excluded for in-situ disease, node negative disease, or unknown cT or cN stage. Another 120,218 patients were excluded for no NAC or unknown receipt of NAC. Lastly, patients who did not undergo axillary surgery or did not have a reported pN stage were excluded. There were 38,559 remaining patients… |
|  |  | (b) Give reasons for non-participation at each stage | 7-8 | *See above* |
|  |  | (c) Consider use of a flow diagram | Fig 1 | Figure 1 |
| Descriptive data | 14* | (a) Give characteristics of study participants (eg demographic, clinical, social) and information on exposures and potential confounders | 22-23 | Table 1 |
|  |  | (b) Indicate number of participants with missing data for each variable of interest | 22-23 | Table 1 |
|  |  | (c) *Cohort study*—Summarise follow-up time (eg, average and total amount) | N/A |  |
| Outcome data | 15* | *Cohort study*—Report numbers of outcome events or summary measures over time | 24-25 | Table 2 |
|  |  | *Case-control study—*Report numbers in each exposure category, or summary measures of exposure | N/A |  |
|  |  | *Cross-sectional study—*Report numbers of outcome events or summary measures | N/A |  |
| Main results | 16 | (*a*) Give unadjusted estimates and, if applicable, confounder-adjusted estimates and their precision (eg, 95% confidence interval). Make clear which confounders were adjusted for and why they were included | 22-27 | Tables 1-3 |
|  |  | (*b*) Report category boundaries when continuous variables were categorized | 22-27 | Tables 1-3 |
|  |  | (*c*) If relevant, consider translating estimates of relative risk into absolute risk for a meaningful time period | N/A |  |

| Other analyses | 17 | Report other analyses done—eg analyses of subgroups and interactions, and sensitivity analyses | Supp Table 2-3 | Supplemental Tables 2-3 |
| --- | --- | --- | --- | --- |
| Discussion | | | | |
| Key results | 18 | Summarise key results with reference to study objectives | 11 | This retrospective cohort study demonstrated facility-level variation in de-escalation of axillary surgery for patients with clinically involved regional nodes who received NAC and had residual axillary involvement, with greatest omission of ALND observed in community facility types. There was overall increased omission of ALND at all facility types over time. Further, facility type effects varied by extent of residual nodal disease. |
| Limitations | 19 | Discuss limitations of the study, taking into account sources of potential bias or imprecision. Discuss both direction and magnitude of any potential bias | 15 | This study is subject to limitations including those inherent to the use of retrospective tumor registry data from participating Commission on Cancer centers. The NCDB does not include patients who are treated outside accredited Commission on Cancer centers. Facility type is suppressed for patients less than 40 years age limiting generalizability of the results about facility types to younger patients. Finally, clinical stage after NAC was not known, and post-NAC staging was inferred from pathologic stage. This is particularly important limitation as decision on surgery is usually made based on clinical nodal status after NAC, which is important when thinking about practice patterns. Additionally, the surgical decisions as result of multi-disciplinary discussion is also unknown. |
| Interpretation | 20 | Give a cautious overall interpretation of results considering objectives, limitations, multiplicity of analyses, results from similar studies, and other relevant evidence | 15-16 | This study evaluated factors associated with omission of ALND in patients with residual nodal disease after NAC undergoing upfront SLNB. While ALND has been de-escalated with a concurrent rise in SLNB alone in this population despite ongoing evaluation of this approach, the odds of omission of ALND were greatest at non-Academic Cancer Centers. The Alliance A011202, ADARNAT, and TAXIS clinical trials will provide additional insight on how to treat the axilla for patients with residual axillary disease after NAC. Until then, current evidence should be weighed in a multidisciplinary discussion along with patient-provider shared decision making to determine the best treatment course for the patient and ensure evidence-based recommendations are appropriately applied. |
| Generalisability | 21 | Discuss the generalisability (external validity) of the study results | 15-16 | *See above* |
| Other information | |  | | |
| Funding | 22 | Give the source of funding and the role of the funders for the present study and, if applicable, for the original study on which the present article is based | 1 |  |

*Give information separately for cases and controls in case-control studies and, if applicable, for exposed and unexposed groups in cohort and cross-sectional studies.

**Note:** An Explanation and Elaboration article discusses each checklist item and gives methodological background and published examples of transparent reporting. The STROBE checklist is best used in conjunction with this article (freely available on the Web sites of PLoS Medicine at http://www.plosmedicine.org/, Annals of Internal Medicine at http://www.annals.org/, and Epidemiology at http://www.epidem.com/). Information on the STROBE Initiative is available at www.strobe-statement.org.
